# Premenstrual Syndrome and Premenstrual Dysphoric Disorder’s Impact on Quality of Life, and the Role of Physical Activity

**DOI:** 10.3390/medicina59112044

**Published:** 2023-11-20

**Authors:** Fabrizio Liguori, Emma Saraiello, Patrizia Calella

**Affiliations:** Department of Medical, Movement Sciences and Wellbeing, University of Naples “Parthenope”, 80133 Naples, Italy; fabrizio.liguori@studenti.uniparthenope.it (F.L.); emmsaraiello@collaboratore.uniparthenope.it (E.S.)

**Keywords:** premenstrual syndrome, quality of life, physical activity

## Abstract

Most women who menstruate experience various physical, psychological, and behavioral changes during the period between ovulation and menstruation. This study focuses on defining and diagnosing premenstrual disorders, distinguishing between premenstrual symptoms (PS), PMS, and premenstrual dysphoric disorder (PMDD). It highlights the prevalence of these conditions and their impact on women’s quality of life, including social, occupational, and psychological aspects. Furthermore, the study examines the role of physical activity, particularly aerobic exercise, in managing premenstrual symptoms. Several systematic reviews are cited, suggesting that regular physical activity can effectively reduce both physical and psychological symptoms associated with PMS. In conclusion, the management of PMS involves a multifaceted approach, with exercises, dietary modifications, stress management, cognitive-behavioral therapy, and medications all playing roles. Physical activity, especially aerobic exercise, has been found to be an effective non-pharmacological therapy for alleviating PMS symptoms and improving overall well-being. However, more research is needed to determine the optimal type and dosage of exercise for individual women with PMS.

## 1. Brief History

Most women who menstruate regularly undergo a range of physical, psychological, and behavioral changes during the period between ovulation and menstruation. One of the first descriptions of these changes was presented by Horney in 1931 [1]. Around the same time as Horney’s description, another significant paper by Frank is often acknowledged as the first modern clinical account of premenstrual symptoms [2]. Like Horney, Frank also used the term “premenstrual tension” to highlight the cyclical emotional disturbances that occurred during the latter part of the menstrual cycle. Although these two researchers began to focus on menstruation and its associated symptoms during the same historical period, their perspectives on this condition were markedly different. The feminist psychoanalyst Karen Horney described “premenstrual tension” as a psychological response to anxieties and fantasies associated with pregnancy, as well as frustrations resulting from cultural restrictions on the expression of female sexuality, while Robert Frank, the gynecologist often credited with identifying premenstrual tension, attributed the symptoms to accumulations of the female sex hormone estrogen and advocated medical intervention. Frank considered premenstrual tension a dysfunction, Horney argued that it was not a pathological process because mood fluctuations, anxiety and irritability, occurred in otherwise healthy women. Frank’s focus shifted increasingly towards a substantial cohort of women facing diverse premenstrual disturbances. It is a well-known fact that ordinary women experience varying levels of discomfort before the onset of menstruation. These minor disruptions encompass heightened fatigue, irritability, diminished concentration, and episodes of pain. However, in a different subset of patients, the reported symptoms were serious enough to necessitate a day or two of bed rest. Among this group, pain takes center stage as the prevailing issue. Yet, another category of patients presented significant systemic disorders during the premenstrual phase. And it is precisely these latter two groups of patients that Frank aimed to shed light on, primarily from a hormonal and clinical perspective, thus laying the groundwork for an in-depth study of these disorders. However, in 1953, Greene and Dalton reasoned that emotional tension was just one of several components of this condition. They suggested that it should be more appropriately named “premenstrual syndrome” [3]. The shift from “premenstrual tension” to “premenstrual syndrome” reflects a transformative evolution in the conceptualization and understanding of the cluster of symptoms experienced by menstruating women. Originally characterized as “premenstrual tension”, the term emphasized the emotional and psychological aspects of the disturbances occurring in the latter part of the menstrual cycle. However, as subsequent research expanded the scope and recognition of diverse physical, emotional, and behavioral changes during this phase, it became evident that the term “tension” inadequately captured the complexity of the condition. This shift allowed for a more inclusive and comprehensive understanding of the condition, recognizing its multifaceted nature.

Furthermore, the transition from “premenstrual tension” to “premenstrual syndrome” not only reflects the evolution in our understanding of women’s experiences, but also underscores the ongoing importance of unravelling the complexities of these symptoms in the context of contemporary health and well-being. Recognizing the diverse nature of premenstrual challenges is not just a historical footnote, but a crucial step towards fostering a more inclusive approach to women’s health in the present day.

## 2. Definition and Diagnosis

Premenstrual disorders refer to psychiatric or physical symptoms that arise during the luteal phase of the menstrual cycle, affecting the individual’s normal daily functioning, and typically subside shortly after menstruation begins. The luteal phase begins after ovulation and lasts until the onset of menstruation.

It is necessary to make a distinction between premenstrual symptoms (PS), premenstrual syndrome (PMS), and premenstrual dysphoric disorder (PMDD).

Typical PS encompass a variety of physical, emotional and behavioral changes, including feelings of depression, angry outbursts, irritability, crying spells, anxiety, confusion, social withdrawal, poor concentration, sleep disturbances, and alterations in thirst and appetite. Additionally, physical symptoms such as breast tenderness, bloating, weight gain, headaches, swelling of the hands or feet, and aches or pains are commonly experienced [4]. In the two weeks prior to the start of menses, up to 75% of premenopausal women are thought to suffer at least one physical or emotional symptom [5].

Between 50 and 80 percent of women feel menstrual discomfort during this time, while between 30 and 40 percent of women develop PMS [6].

PMS has been categorized by the World Health Organization (WHO) under the ICD-10, the 10th iteration of the International Classification of Diseases [7]. Any woman of childbearing age may suffer from this illness; anxiety, physical aches, breast soreness, and irritability are the most prevalent symptoms [8]. According to the American College of Obstetrics and Gynecology, PMS is the cyclical occurrence of symptoms that are severe enough to affect certain elements of life and that occur in a predictable and consistent manner in relation to the menses [9]. Anytime between menarche and menopause, symptoms may appear. The cost of the illness can be significant; women with PMS miss more work, pay more for healthcare, and have lower health-related quality of life [10].

PMS can be diagnosed if woman reports at least one of the affective and somatic symptoms, defined by the American College of Obstetricians and Gynecologists, during the five days before menses in each of the three previous menstrual cycles.

PMDD affects 3–8% of women, and is a severe version of PMS. The extreme variant of PMS known as PMDD mostly manifests psychological symptoms [11]. The *Diagnostic and Statistical Manual of Mental Disorders*, Fifth Edition (DSM-5) now includes PMDD as a new diagnostic category for depression, according to the American Psychiatric Association [12]. Bipolar disorder and generalized anxiety disorder are two conditions that are more prevalent in PMS-affected women. According to a meta-analysis, women with PMDD have a fourfold higher risk of suicidal thoughts and a sevenfold higher risk of suicidal attempts [13].

Even for the PMDD, as for the PMS, to meet the diagnostic criteria, a patient must have at least five of the symptoms listed by the America Psychiatric Association in the week before menses, and these symptoms must improve within a few days after the onset of menses [14].

The best technique to identify PMS and PMDD is using prospective surveys since individuals vastly overestimate the cyclical pattern of symptoms when actually they are erratic or merely made worse during the luteal phase. PMS or PMDD can be identified using the “Daily Record of Severity of Problems”, a valid and trustworthy diagnostic instrument, which is a daily record of symptoms that correspond to the PMS and PMDD diagnostic standards [15].

PMDD represents a severe and, at times, incapacitating extension of PMS. While both PMS and PMDD entail physical and emotional symptoms, PMDD is characterized by intense mood fluctuations that have the potential to disrupt daily functioning and strain interpersonal relationships. In both PMDD and PMS, symptoms typically manifest seven to ten days before the onset of menstruation, persisting into the initial days of the menstrual period. Common features of both PMDD and PMS encompass bloating, breast tenderness, fatigue, and alterations in sleep and dietary patterns. However, PMDD is distinguished by the prominence of at least one emotional or behavioral symptom, which may include: feelings of sadness or hopelessness, anxiety, intense mood swings, and pronounced irritability or anger. Table 1 showed the main differences and commonalities between PMS and PMDD.

## 3. Epidemiology and Etiology

During the luteal phase of their menstrual cycle, almost 80% of women report at least one physical or psychological symptom; however, the majority do not report a severe impairment in their everyday lives. Age, educational attainment, or employment status have no bearing on the frequency of PMS. The duration and severity of symptoms can change. Only 36% of women who were given a PMS diagnosis remained to match the criteria a year later, according to one study [21]. Women who gained weight or experienced a stressful incident in the last year are more likely to be diagnosed with PMS. The prevalence of PMDD ranges from 1.3% to 5.3%, and fewer women match the stricter diagnostic standards [22].

In a Japanese study, a significant number of Japanese women between the ages of 20 and 49 years, who attended a clinic for female cancer screening, reported premenstrual symptoms, and the prevalence rates of moderate and severe PMS and PMDD were 5.3% and 1.2%, respectively [23]. The 2007 Swiss Nationwide Health Survey revealed that 91% of Swiss women aged 15–54 years reported experiencing at least one symptom. Among them, 10.3% were diagnosed with PMS, while 3.1% met the criteria for PMDD [24].

In a Chinese population-based study involving women aged 18–45 years, the prevalence of PMDD was 2.1%, and that of PMS was 21.1% [25]. A meta-analysis that systematically reviewed the prevalence of PMS highlighted as the pooled prevalence of PMS was 47.8% worldwide, with the lowest prevalence reported in France (12%) and highest in Iran (98%) [26]. Future studies must consider the prevalence of PMS in various nations of the world, because different techniques have been utilized in studies and many have been designed based on a small sample.

The cause of PMS is complex and may be influenced by a combination of hormonal, genetic, environmental, and sociocultural factors. Additionally, other aspects related to the menstrual cycle, such as the age of menarche, menstrual flow, and other menstruation-related disruptions, may also contribute to the development of PMS [27]. The exact cause of premenstrual disorders remains poorly understood. Some research indicates that cyclical changes in estrogen and progesterone levels may trigger the symptoms [28]. Postmenopausal women with a history of PMS experienced recurring psychiatric and physical symptoms when undergoing cyclical progestogen therapy, suggesting a hormonal influence. Suppression of estrogen using gonadotropin-releasing hormone analogues has also shown significant improvement in PMS symptoms [29].

Moreover, the reasons why some women may be more sensitive to hormonal fluctuations are not yet well understood [30].

Genetics may also play a role in premenstrual disorders, as suggested by monozygotic twin studies. However, specific genes responsible for these conditions have not been identified yet [31]. Overall, the etiology of premenstrual disorders involves a complex interplay of hormonal, genetic, and possibly other factors that require further investigation.

## 4. PMS, PMDD, and Quality of Life

A study by Kathleen et al. performed in a sample of young women found that, compared to women with low PMS, women with high PMS reported much more stress and a lower quality of life [32]. According to these findings, various studies show that PMS severely lowers patients’ quality of life and places a heavy load on their ability to do their regular daily responsibilities and activities [33]. One study that examined the efficacy of a psycho-educational PMS intervention found that while it reduced the intensity of PMS and its associated somatization, anxiety, and hostility, it had no effect on the degree of sadness or interpersonal sensitivity [34].

PMS can significantly interfere with social life, employment, school, interpersonal relationships, and family [35,36], and is associated with decreased occupational productivity, poorer perceived quality of sleep, and increased healthcare use [37].

PMDD is a severe, sometimes disabling, extension of PMS, causing extreme mood shifts that can disrupt work and damage relationships [11]. In addition, PMS and PMDD may result in anxiety, depressed mood, and greater psychiatric comorbidity, producing both direct and indirect medical costs due to absenteeism and low productivity [38].

Some experts have proposed that a considerable number of women experiencing premenstrual symptoms might not be entirely absent from work; instead, they may choose to reduce their working hours or find their work efficiency to be diminished during this time. This implies that even though they may be physically present at their workplaces, their ability to perform optimally might be affected by the symptoms they are experiencing. It is crucial to recognize that the impact of premenstrual symptoms on women’s work performance can vary widely, and while some may opt for complete absence, others may still be affected to some extent, even when present at work. Therefore, addressing the issue of premenstrual symptoms in the workplace goes beyond just considering the absence of affected women, but also involves understanding the potential productivity challenges they may encounter during this phase of the menstrual cycle. Employers and organizations should be aware of these possibilities and consider providing support and accommodations to ensure the well-being and productivity of their female employees during such times [39].

Numerous studies have established a link between PMS and stress [40,41]. Brown and Lewis specifically investigated perceived stress levels in women with varying degrees of menstrual symptomatology; their findings indicated that the high PMS group experienced significantly more hassles (a stress indicator) and fewer uplifts (positive experiences) before menstruation compared to after menstruation [41].

These results suggest that PMS might be a condition influenced by stress, emphasizing the need for stress-reduction treatments. One potential behavioral intervention to reduce stress is physical activity.

## 5. Physical Activity

According to numerous researchers, different physiological systems, including the cardiovascular, central nervous system, endocrine, and female reproductive system, have been linked to PMS symptoms in terms of emotional, physical, cognitive, and behavioral aspects. Various treatment options are recommended for managing PMS symptoms. For instance, both the National Institute for Health and Care Excellence (NICE) and the Royal College of Obstetricians and Gynaecologists (RCOG) advocate exercise as a primary intervention. Additionally, medications like selective serotonin reuptake inhibitors and the combined oral contraceptive pill are also suggested alongside exercise [42]. Nevertheless, physical exercise is recognized for its ability to elevate endorphin levels, regulate the synthesis of progesterone and estrogen, and stimulate the production of naturally occurring anti-inflammatory substances [43]. In addition, exercise offers various other advantages, including enhanced overall fitness, opportunities for social interaction, and the potential to alleviate feelings of depression. These combined benefits may contribute to moderating the range of symptoms experienced in PMS [44]. A recent systematic review screened 15 randomized controlled trials comparing exercise interventions of a minimum of 8-weeks duration with non-exercise comparator groups in women with PMS, and highlighted that exercise may be an effective treatment for PMS [45]. They found that engaging in physical exercise could be beneficial in reducing psychological, physical, and behavioral symptoms linked to PMS, and may aid in managing the overall range of symptoms experienced during this period. These findings are consistent with those from another systematic review that investigated the effects of any form of physical exercise in women with PMS; the studies included effect of exercise such as aerobic exercise, yoga, swimming, and Pilates. Regardless of the specific type of exercise, regular physical activity appears to be effective in alleviating pain, constipation, and breast sensitivity, as well as psychological symptoms such as anxiety and anger, but the most effective type of exercise remains uncertain. As the exercise types seem to have similar effects on symptoms, individuals might be encouraged to choose the type of exercise that suits them best. Furthermore, engaging in long-term regular exercise programs is the crucial factor for a favorable outcome [46].

The most recent systematic review on the topic focused on the impact of aerobic exercise in women of reproductive age with PMS, aimed to assess whether aerobic exercise is effective in improving premenstrual symptoms in women of reproductive age [47]. Only five studies meet the inclusion criteria, with the main findings that engaging in 30 min of aerobic exercise, three to five times a week, appears to be effective in reducing physical PMS symptoms. The statistical results from the included studies indicate that aerobic exercises have a greater impact on improving physiological symptoms compared to psychological symptoms in women with PMS.

In conclusion, the management of PMS involves a combination of approaches, including exercises, dietary modifications, stress management, cognitive-behavioral therapy, and the use of medications based on specific symptoms. According to the guidelines provided by the American College of Obstetricians and Gynecologists (ACOG), non-pharmacological therapies are recommended as the primary treatment for all women with PMS [48].

## 6. Conclusions and Future Directions

Physical activities are regarded as a beneficial alternative to medications for managing premenstrual symptoms, and they have been associated with promoting well-being during PMS episodes. Engaging in regular physical activities can potentially improve hormonal balance, reproductive function, menstrual cyclicity, ovulation, and fertility in women of all ages [49]. Scientific evidence from literature indicates that exercises during PMS can lead to improvements in hematological parameters and a reduction in the levels of certain hormones such as prolactin, estradiol, and progesterone [50].

Moreover, various studies have found that exercises can contribute to enhancing self-esteem, reducing depression, and alleviating anxiety among women experiencing PMS. 

Previous reviews in the literature that recommend exercise during PMS have considered a wide range of physical exercises, including yoga, Pilates, and strength and conditioning. While exercises have been shown to be beneficial for managing PMS, there is still uncertainty about the specific type and dosage of therapeutic exercises that are most suitable for females with PMS. Additionally, there is a lack of specific reviews focusing on the impact of aerobic exercise in women of reproductive age with PMS. Therefore, the objective of this systematic review is to assess whether aerobic exercise is effective in improving premenstrual symptoms in women of reproductive age.

In summary, the management of PMS involves a multi-faceted approach, with exercises playing a significant role as a non-pharmacological therapy. While exercise has shown positive effects in improving certain symptoms and overall well-being during PMS, further research is needed to better understand its impact on different individuals and to develop personalized exercise recommendations for women experiencing premenstrual symptoms.

The take-home messages are as follows:

- High PMS levels correlate with increased stress and lower quality of life;
- PMDD, a severe extension, disrupts work and relationships, correlating with psychiatric comorbidity and increased medical costs;
- Exercise is recommended for managing PMS symptoms, offering benefits like improved hormonal balance and psychological well-being;
- Aerobic exercise, 30 min, 3–5 times weekly, shows effectiveness in reducing physical PMS symptoms;
- Non-pharmacological therapies, including exercise, are primary treatments per ACOG guidelines;
- Future research should focus on personalized exercise recommendations for women with premenstrual symptoms.

## Figures and Tables

**Table 1:** Comparison of premenstrual syndrome (PMS) and premenstrual dysphoric disorder (PMDD): key characteristics and distinctions.

	Premenstrual Syndrome	Premenstrual Dysphoric Disorder
Definition	PMS refers to a group of physical and behavioral symptoms that occur in a cyclic pattern during the second half of the menstrual cycle.	PMDD is a neuro-hormonal gynecological disorder. It is the severe form of PMS.
Signs and symptoms	Variations in appetite, gaining weight, discomfort in the abdomen, pain in the back (especially the lower back), headaches, breast swelling and sensitivity, feelings of nausea, constipation, heightened anxiety, irritability, anger, fatigue, restlessness, mood fluctuations, and episodes of crying.	PMDD symptomatology encompasses a multifaceted array of mood-related, behavioral, and somatic manifestations: mood lability, sadness, anxiety, lack of energy and persistent fatigue, changes in appetite, joint or muscle aches, and a sensation of bloating or weight gain.
Diagnostic criteria	There is no test for PMS or PMDD. To be diagnosed with PMS or PMDD, a woman must have the symptoms that must occur before her menstrual period and disappear after the onset of the period.	It is classified in the DSM-5-TR as a mental illness. The criteria for PMDD require that the woman experience at least 5 of 11 cognitive-affective, behavioural, and physical symptoms during the final week of the luteal phase that resolve with or near the onset of menses. Symptoms must also remit post-menses, and not represent an exacerbation of another psychiatric disorder.
Epidemiology Worldwide	47.8% [16].	1.2–6.4%
Etiology	The exact etiology is not known	The exact etiology is not known
Risk factors	The primary risk factors associated with PMS and PMDD included a negative rhesus blood type, the age of menarche, intake of caffeine, and self-reported depression [17].	Traumatic events and pre-existing anxiety disorders are risk factors for the development of PMDD [18].
Specific genes	Recent research has offered evidence supporting the participation of the gene responsible for coding the serotonergic 5HT1A receptor and allelic variations in the estrogen receptor alpha gene (ESR1) at the onset of PMS/PMDD [19,20].	Recent research has offered evidence supporting the participation of the gene responsible for coding the serotonergic 5HT1A receptor and allelic variations in the estrogen receptor alpha gene (ESR1) at the onset of PMS/PMDD [19,20].
Managment	The use of a combination of medications (such as anxiolytics, gonadotropin-releasing hormone agonists, spironolactone, and oral contraceptive pills) alongside nonpharmacological approaches, primarily involving cognitive and behavioral therapies, exercises, massage therapy, light therapy, and dietary/nutritional adjustments, has demonstrated effectiveness in treating premenstrual symptoms.	Treatment modalities for PMDD can be divided into two groups: non-pharmacological methods as exercise, dietary modifications, stress management with relaxation, meditation and breathing techniques. Pharmacological methods are psychotropic agents and hormonal therapies for suppression of ovulation.
Suicidability	There was no significant association between PMS and suicide attempts (OR: 1.85; 95% CI: 0.77–4.46, *p* = 0.17). Women with PMS are at increased risk of suicidal ideation, but not suicide attempts [13].	PMDD diagnosis increased the risk of suicide attempts by approximately sevenfold (OR: 6.97; 95% CI: 2.98–16.29, *p* < 0.001). PMDD diagnosis increased the risk of suicidal ideation by approximately fourfold (OR: 3.95; 95% CI: 2.97–5.24, *p* < 0.001) [13].

## Data Availability

Data are contained within the article.
